# Xingkai Lake Topmouth Culter (*Culter alburnus*) Exhibits Biochemical and Histopathological Alterations upon Acute Ammonia Exposure

**DOI:** 10.3390/antiox14111318

**Published:** 2025-10-31

**Authors:** Junfei Yu, Hongling Yang, Guohe Cai, Jianming Xu, Banghua Xia, Yunzhang Sun

**Affiliations:** 1 Fisheries College, Jimei University, Xiamen 361021, China; 202514908002@jmu.edu.cn (J.Y.); 200561000133@jmu.edu.cn (H.Y.); caiguohe@jmu.edu.cn (G.C.); 202214908006@jmu.edu.cn (J.X.); 2 Marine College, Shandong University, Weihai 264209, China

**Keywords:** *Culter alburnus*, ammonia exposure, oxidative stress, cellular apoptosis

## Abstract

The Xingkai Lake topmouth culter (*Culter alburnus*) is an endemic, economically valuable fish in Heilongjiang that is highly sensitive to ammonia. However, the systemic effects of acute ammonia stress on its liver have not been determined. The objective of this study was to elucidate the changes in and relationships among stress biomarkers, antioxidant defense mechanisms, apoptosis indicators, and histopathological alterations in the liver of *C. alburnus*, a fish species native to Xingkai Lake, China, under acute ammonia exposure. Guided by the findings of a 96 h-LC_50_ assay, the researchers subjected the fish to 48 h of acute exposure at specified total ammonia nitrogen (TAN) concentrations of 30 mg/L, 36 mg/L, and 40 mg/L. A comprehensive assessment of physiological and biochemical markers, including cortisol (COR), blood ammonia (Amm), blood glucose (Glu), aspartate aminotransaminase (AST), alanine aminotransaminase (ALT), catalase (CAT), superoxide dismutase (SOD), glutathione peroxidase (GSH-Px), and malondialdehyde (MDA), revealed pronounced physiological stress and oxidative damage, particularly in the high-concentration groups. The physiological effects of ammonia exposure on *C. alburnus* showed a clear concentration and time dependence. Notably, elevated ammonia levels significantly upregulated apoptosis-associated genes such as *P53*, *Bax*, and *Caspase-3*. These findings were further substantiated by terminal deoxynucleotidyl transferase-mediated dUTP nick end labeling (TUNEL) assays and histopathological examinations. Overall, the study demonstrated that acute ammonia exposure exerted substantial impacts on the physiological, biochemical, and genetic expression profiles of *C. alburnus* in Xingkai Lake, leading to sustained stress and oxidative damage, especially at elevated concentrations (30–40 mg/L).

## 1. Introduction

The topmouth culter (*Culter alburnus*) belongs to the order Cypriniformes, family Cyprinidae, and genus Culter. It is a highly valued economic fish endemic to regions such as the middle and lower reaches of the Yangtze River and the Heilongjiang River Basin [1]. The Xingkai Lake whitefish represents a distinct geographical population within this species [2]. Also known as the Xingkai Lake whitefish, it is celebrated as one of the “Four Major Freshwater Delicacies” in China, alongside the Songjiang perch (*Trachidermus fasciatus*), the Yellow River carp (*Cyprinus carpio*), and the Songhua mandarin fish (*Siniperca chuatsi*). Renowned for its delicate flavor, tender texture, and high nutritional value, the Xingkai Lake whitefish has attracted significant attention from both consumers and researchers [3]. With growing imperatives for resource conservation and sustainable utilization, the industry model for Xingkai Lake whitefish has progressively shifted over the past two decades from reliance on wild stock capture to artificial cultivation. During this period, researchers have conducted systematic fundamental studies on its biological traits, artificial breeding technologies, nutritional requirements and feed optimization, and the regulation of flesh quality attributes [4,5,6,7]. Concurrently, aquaculture practices have diversified to include traditional pond culture, intensive high-density culture, and extensive stock enhancement in large- and medium-sized water bodies, adapting to various production contexts and ecological conditions [8].

The emergence of high-density intensive aquaculture models has led to the continuous accumulation of residual feed and excreta in aquaculture water bodies. After decomposition by bacteria, these substances cause a sharp increase in ammonia concentration in the water. High concentrations of ammonia exert significant toxicological effects on aquatic ecosystems, with particularly pronounced impacts on fish species, inducing a range of adverse consequences, including enzyme disorders, organ lesions, and oxidative stress, among others [9,10]. Initial exposure to ammonia was found to compromise the hepatic detoxification pathways in fish [11,12]. Subsequent research delineated that such exposure modulated the levels of key metabolic enzymes, including glutamate transaminase (GPT), glucose (Glu), and glutamate oxaloacetate transaminase (GOT), with the magnitude of these alterations contingent upon the exposure duration and ammonia concentration [13]. Moreover, ammonia exposure instigated oxidative stress within the hepatic tissues, disrupting the redox equilibrium through the excessive generation of reactive oxygen species (ROS). Intriguingly, fish exhibited the capability to adjust the activities of both enzymatic and non-enzymatic antioxidants, such as glutathione (GSH), to counteract ROS-mediated oxidative damage [14]. Lastly, ammonia exposure was implicated in modulating hepatic immune responses, evidenced by the upregulation of pro-inflammatory cytokines integral to the innate immune system [15]. Consequently, fish, which are foundational to the aquatic food web, are considered sensitive bioindicators for environmental pollutants. While a considerable body of literature has been dedicated to exploring the ramifications of oxidative stress on aquatic organisms [16,17,18], studies focusing on the use of Xingkai Lake whitefish as a bioindicator for ammonia exposure remain scarce, and its adaptive mechanisms and tolerance thresholds are still poorly understood.

This study provides substantive experimental evidence for developing specific biomarkers in *C. alburnus* as high-sensitivity early warning tools, thereby addressing a significant knowledge gap in the ecotoxicology of ammonia nitrogen for this species. Through a systematic analysis of stress biomarkers, antioxidant defense, apoptosis, and histopathological alterations following acute ammonia exposure, this work is the first to integrate the multi-level responses from physiological stress to tissue damage, thereby elucidating the underlying toxic pathway mechanism. The findings not only confirm the pivotal role of oxidative stress as a core injury mechanism but also, more importantly, establish a causal relationship between ammonia nitrogen exposure and cellular death coupled with organ dysfunction by correlating apoptotic indicators with histopathological changes.

## 2. Materials and Methods

### 2.1. Experimental Materials

In June 2024, a total of 750 *C. alburnus* (an artificially cultured population from the Heilongjiang Nongken Zhenda Xingkai Lake Whitefish Research Institute) specimens of uniform size and free from any visible injuries (190 ± 10 g each) were collected from the Xingkai Lake basin in Jixi City, Heilongjiang Province, China (N 132°22′10″, E 45°47′43″). The fish were transported in oxygenated bags to the Heilongjiang Fisheries Research Institute of the Chinese Academy of Fishery Sciences (Harbin, China). Upon arrival, *C. alburnus* were acclimated for 14 days in recirculating tanks (100 cm × 50 cm × 50 cm) with an effective water volume of 200 L, at a density of 10 fish per tank (*n* = 10). The experimental water temperature was maintained at 16 ± 0.5 °C, dissolved oxygen (DO) levels at 6.8 ± 0.2 mg/L, and pH at 7.78, with total ammonia levels below 0.05 mg/L. A 12 h:12 h light-dark cycle was employed. Fish were fed commercial feed to satiation daily, and feeding was ceased 48 h prior to the start of the experiment. Ammonia nitrogen was sourced from NH_4_Cl (CAS: 12125-02-9, purity ≥ 99.5%) supplied by Sinopharm Chemical Reagent Co., Ltd., Shanghai, China. A precisely weighed 38.19 g of NH_4_Cl was dried to constant weight and diluted to 1 L to prepare a 10 g/L NH_4_Cl stock solution, which was subsequently used for adjusting total ammonia concentrations in the water. The animal study protocol was approved by the Animal Ethics Committee of Jimei University, Xiamen, China (Approval No.: 2021-04, 22 January 2021).

### 2.2. Determination of LC_50_

Ammonia nitrogen concentrations were set at logarithmic intervals, ranging from the lowest concentration with no mortality within 96 h (23 mg/L) to the highest concentration where all fish perished (56 mg/L). Total Ammonia Nitrogen (TAN) concentrations were set at logarithmically equidistant intervals of 26.68 mg/L, 30.94 mg/L, 35.89 mg/L, 41.63 mg/L, and 48.28 mg/L. The 96 h-LC_50_ was calculated based on these results [19]. During the preliminary static water culture, three replicates were established for each group, each containing 10 fish. One-third of the water volume was replaced daily, and total ammonia nitrogen concentrations were measured every 8 h using Nessler’s reagent method, calibrated using a pre-prepared NH_4_Cl stock solution.

### 2.3. Experiment of Acute Ammonia Exposure Design

Based on the 96 h-LC50 results, the experimental design comprised four treatment groups: control (CK, 0 mg/L), low concentration (25% mortality, 30 mg/L), medium concentration (50% mortality, 36 mg/L) and high concentration (75% mortality, 40 mg/L). Three replicates were established for each group, each containing 50 fish. Waterborne ammonia concentrations were monitored every 8 h throughout the exposure period using Nessler’s reagent method, with concentrations verified and adjusted as necessary using a freshly prepared ammonia stock solution to maintain exposure accuracy. Tissue and blood samples were collected at six predetermined time points (0, 3, 6, 12, 24, and 48 h) post-exposure. At each interval, six fish per group were randomly selected and anesthetized with 250 mg/L MS-222 (Sigma, Ronkonkoma, NY, USA). Blood was drawn from the caudal vein using a 1 mL medical syringe and held at 4 °C for temporary storage. Following euthanasia, liver tissues were promptly dissected. Two-thirds of each liver sample was flash-frozen in liquid nitrogen and stored at −80 °C for subsequent physiological, biochemical, and gene expression analyses. The remaining one-third was fixed in 4% paraformaldehyde (Sinopharm Chemical Reagent Co., Ltd., Shanghai, China) for histopathological examination.

### 2.4. Biochemical Analysis

Collected blood was allowed to stand at 4 °C for 12 h, after which serum was collected by centrifugation at 3500× *g* for 10 min at 4 °C using a low-temperature centrifuge (iCEM-24, Aosheng Instrument Co., Ltd., Hangzhou, China). Serum was stored at −80 °C and analyzed for cortisol (COR), blood ammonia (Amm), blood glucose (Glu) contents, aspartate aminotransaminase (AST), and alanine aminotransaminase (ALT) activities according to the manufacturer’s instructions (Nanjing Jiancheng Bioengineering Institute, Nanjing, China). Liver tissues stored at −80 °C were slowly thawed and homogenized in physiological saline at 4 °C at a weight-to-volume ratio of 1:9 (g/mL). The homogenate was then centrifuged at 3000× *g* for 20 min at 4 °C, and the supernatant was collected. Catalase (CAT), superoxide dismutase (SOD), glutathione peroxidase (GSH-Px) activities, and malondialdehyde (MDA) level, as well as total protein concentration (TP), were determined according to the manufacturer’s instructions (Nanjing Jiancheng Bioengineering Institute, Nanjing, China) [20].

### 2.5. Gene Expression Analysis

Total RNA was extracted from liver tissues using the BioFast^®^ Simply P^®^ Total RNA Analysis Kit (BIOER, Hangzhou, China) and the concentration and quality of the RNA were verified using a Uv-Vis spectrophotometer NanoPhotometer N60 Touch (Implen, München, Germany). The isolated RNA was stored at −20 °C prior to use. Complementary DNA (cDNA) was synthesized using the BioRT^®^ Master HiSensi^®^ cDNA First Strand Synthesis Kit (BIOER, Hangzhou, China), and its concentration and quality were also confirmed using the aforementioned spectrophotometer. Quantitative PCR (qPCR) reactions were set up in a 10 μL volume, comprising 5 μL of 2 × SYBR Green PCR Master Mix, 0.2 μL of each primer, 1.0 μL of cDNA, and 3.6 μL of RNase-free water. The genes evaluated are listed in Table 1. Following reagent mixing, qPCR was performed under the following conditions: an initial denaturation at 95 °C for 60 s; 35 cycles of denaturation at 95 °C for 15 s and annealing at 60 °C for 60 s; and a melt curve analysis at 95 °C for 60 s, 65 °C for 60 s, and 95 °C for 20 s (increment of 0.5 °C/s), followed by a final hold at 30 °C for 60 s. Beta-actin was employed as the internal reference gene to normalize all samples. The relative changes in mRNA expression were calculated using the 2^−ΔΔCT^ method, and the specificity and non-specificity of the products were distinguished through melt curve analysis. Primer efficiency (%) and R^2^ were determined via standard curve analysis following the Minimum Information for Publication of qPCR Experiments (MIQE) guidelines. Efficiency values range from 94.5% to 102.2% (optimal range: 90–110%), and R^2^ > 0.99, confirming primer pair specificity and amplification reliability. The qPCR reactions were conducted in triplicate, and data from all three replicates were collected for statistical analysis [21].

### 2.6. Terminal Deoxynucleotidyl Transferase-Mediated dUTP Nick End Labeling Assay (TUNEL)

Liver tissue sections were subjected to TUNEL assays using the Fluorescein TUNEL Cell Apoptosis Detection Kit (Servicebio, Wuhan, China). The methodology was adapted from [22] with modifications. Briefly, liver tissue sections were deparaffinized and treated with 20 μg/mL proteinase K at 37 °C for 15 min, followed by three washes with PBS. The sections were then incubated in the dark at 37 °C for 60 min with the prepared TUNEL detection solution. After three additional PBS washes, apoptotic cells were visualized under a fluorescence microscope with an excitation wavelength range of 450–500 nm and an emission wavelength range of 515–565 nm (green fluorescence). DAPI staining was performed for 10 min, and cell nuclei were observed under a fluorescence microscope with an excitation wavelength range of 360–400 nm and an emission wavelength of 460 nm (blue fluorescence).

Quantitative analysis of the obtained immunofluorescence images was conducted as follows: For each group, 3 biological replicate samples were randomly selected. From the section of each sample, 5 non-overlapping fields of view (×200 magnification) were randomly chosen. The total number of cells stained with DAPI (blue fluorescence) and the number of TUNEL-positive apoptotic cells (green fluorescence) in each field of view were counted separately. The apoptosis rate was calculated using the formula: Apoptosis rate (%) = (Number of TUNEL-positive cells/Total number of cells) × 100%. All counting operations were performed by 2 experimenters in a double-blind manner to reduce subjective errors.

### 2.7. Histopathology

Isolated liver tissues were rinsed with physiological saline and fixed in 4% paraformaldehyde for 48 h. Tissue blocks measuring 10 mm × 10 mm × 5 mm were prepared and subjected to a series of gradient ethanol (CAS: 64-17-5, purity ≥ 99.8%) and xylene (CAS: 1330-20-7, purity ≥ 99%) treatments for dehydration and clearing, respectively. Following paraffin embedding (CAS: 8002-74-2), sections were stained with hematoxylin and eosin (H&E) and sealed with neutral resin. Observations and photographic documentation were conducted under an optical microscope (Olympus, Tokyo, Japan).

### 2.8. Statistics

Experimental results were analyzed using SPSS Statistics 20.0.0 (IBM Corporation, Armonk, NY, USA). Prior to parametric tests, data normality was evaluated via the Shapiro–Wilk test and homoscedasticity via Levene’s test. For physiological and biochemical indicators, a two-way ANOVA was employed to evaluate differences with ammonia exposure concentration (control, 30, 36, 40 mg/L TAN) and exposure time (0, 3, 6, 12, 24, 48 h) as fixed factors. This test simultaneously assessed the main effects of each factor independently and their interactive effect. Post hoc comparisons were performed using Duncan’s multiple range test. Cell counting was performed using ImageJ software (Version 1.8.0). TUNEL fluorescence-detected apoptosis rates were subjected to *t*-tests for significance analysis. The 96 h-LC50 was determined via weighted regression analysis, and ELISA data were processed using the Logistic four-parameter model in ELISA Calc (Version 0.1). Data are presented as mean ± standard error (mean ± SE). Graphs were generated using GraphPad Prism 8. Differences were considered statistically significant at *p* < 0.05.

## 3. Results

### 3.1. Determination of 96 h Lethal Concentration (LC_50_) for C. alburnus Following Acute Ammonia Exposure

The concentrations of total ammonia nitrogen (TAN) and non-ionic ammonia (CNH_3_), and the total amount, total deaths and cumulative mortality rate (CMR) of *C. alburnus* observed during the 96 h ammonia exposure pre-trial are presented in Table 2. The calculation formula is as follows: utilizing the CNH_3_ calculation formula, the 96 h-LC_50_ for TAN was determined to be 36 mg/L, and for CNH_3_, it was ascertained to be 2.55 mg/L. Consequently, the safe concentration for aquaculture for CNH_3_ in *C. alburnus* was established at 0.255 mg/L. Based on the pre-trial results, experimental concentrations of total ammonia were set at 0 mg/L for the control group, 30 mg/L for the low-concentration group, 36 mg/L for the medium-concentration group, and 40 mg/L for the high-concentration group, corresponding to mortality rates of 0%, 25%, 50%, and 75%, respectively.

$$\mathrm{Safe}\;\mathrm{concentration}\;(C)=0.1\times 96\;h\text{-}{\mathrm{LC}}_{50}$$

$$\mathrm{Non}\text{-}\mathrm{ionic}\;\mathrm{ammonia}\;({\mathrm{NH}}_{3})=\frac{{{NH}_{4}}^{+}+{\mathrm{NH}}_{3}}{10\;(pKa-pH)+1}$$

$$pKa=0.09018+\frac{2729.92}{T}$$

T=237.15+t

Cumulative Mortality Rate (CMR)=total deaths/total amount

where

NH_4_^+^ + NH_3_: The sum of the concentrations of ammonium ions and nonionic ammonia in water
*pKa*: The negative logarithm of the acid dissociation constant for the ammonia dissociation reaction
pH: Acid-base level of water
T: Absolute temperature; t: Centigrade degree.

### 3.2. Oxidative Stress, Hepatic Function, and Antioxidant Markers in C. alburnus Liver Tissue Following Acute Ammonia Exposure

**Figure 1:**
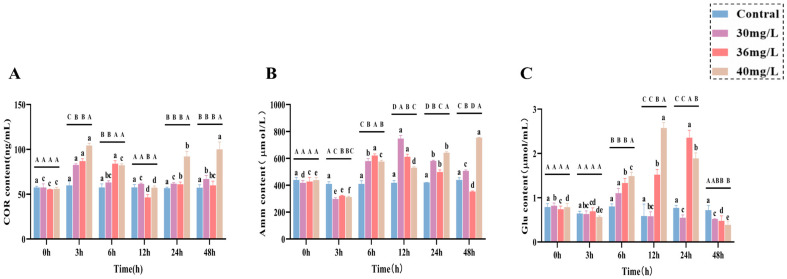
Effects of acute ammonia exposure on stress hormones, metabolites, liver function, and oxidative stress-related markers in liver tissues of *C. alburnus*. (**A**): Cortisol (COR) content; (**B**): Ammonia (AMM) content; (**C**): Glucose (Glu) content. All data are presented as mean ± standard error (*n* = 3 per group). Upper-case letters denote significant differences between groups at the same time point (*p* < 0.05), and lower-case letters indicate significant differences within the same group at different time points (*p* < 0.05).

During the acute ammonia exposure study on *C. alburnus*, significant variations were observed in various physiological and biochemical parameters across different treatment concentrations and time points. These included a stress hormone (COR), metabolites (Amm and Glu), hepatic function markers (AST and ALT), antioxidant enzyme activities (CAT, SOD, and GSH-Px), and oxidative stress markers (MDA). Specifically, it was shown in the following aspects: in terms of stress hormones, the levels of COR (Figure 1A) in the serum of the treated groups all reached the maximum value after 3 h of ammonia exposure and showed a strong concentration dependence. In the low and medium-concentration groups, the COR content returned to the control level after 24 h, whereas in the high-concentration groups the COR content showed a complex fluctuation pattern, but always maintained a relatively high level. Regarding metabolites, the Amm (Figure 1B) content in the treatment group decreased to the lowest value at 3 h post-exposure, which was significantly lower than that in the control group (*p* < 0.05). For the concentration-specific patterns of Amm, the content in the low and medium-concentration groups peaked at 12 h and then gradually decreased; by 48 h, it was significantly lower than that in the high concentration treatment group (*p* < 0.05). Notably, the Amm content in the high concentration treatment group exhibited an overall positive correlation with exposure time after 3 h. For Glu content (Figure 1C), the magnitude was positively correlated with the exposure concentration. Specifically, the low-concentration group reached its peak at 6 h and then declined, the medium-concentration group peaked at 24 h followed by a decline, and the high-concentration group peaked at 12 h before declining.

**Figure 2:**
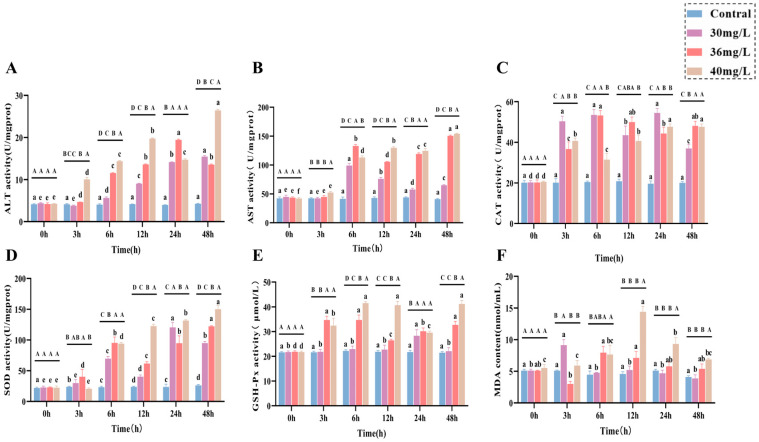
Effects of acute ammonia exposure on stress hormones, metabolites, liver function, and oxidative stress-related markers in liver tissues of *C. alburnus*. (**A**): Aspartate Aminotransferase (AST) activity; (**B**): Alanine Aminotransferase (ALT) activity; (**C**): Catalase (CAT) activity; (**D**): Superoxide Dismutase (SOD) activity; (**E**): Glutathione Peroxidase (GSH-PX) activity; (**F**): Malondialdehyde (MDA) content. All data are presented as mean ± standard error (*n* = 3 per group). Upper-case letters denote significant differences between groups at the same time point (*p* < 0.05), and lower-case letters indicate significant differences within the same group at different time points (*p* < 0.05).

In terms of liver function indexes, both AST (Figure 2A) and ALT (Figure 2B) activities showed an increasing trend and were positively correlated with the exposure concentration. In terms of antioxidant and oxidative stress, CAT, SOD, GSH-Px, and MDA all showed a positive correlation pattern between ammonia exposure time and concentration, in which CAT (Figure 2C) activities were all significantly higher than that of the control group (*p* < 0.05), with a certain degree of fluctuation, and it is worth noting that there was no significant difference between CAT activities of the high-concentration group and those of the medium-concentration group, but they were lower than those of the low-concentration group as a whole. The SOD (Figure 2D) activity was significantly higher in the middle concentration group than in the low-concentration group at 3–12 h and 48 h (*p* < 0.05), whereas SOD activity in the high-concentration group was significantly higher than that in the middle concentration group at both 12–48 h (*p* < 0.05), although at 6 h there was no significant difference between them; and the GSH-Px (Figure 2E) activity in the low-concentration group was higher than that of the control group (*p* < 0.05), while both the medium and high-concentration groups were significantly higher than the control group (*p* < 0.05) throughout the exposure period and showed some degree of fluctuation; the MDA content (Figure 2F) showed a trend of increasing and then decreasing in all treatment groups, with the low-concentration group reaching a peak at 3 h and being significantly higher than the control group (*p* < 0.05), and the medium-concentration group reaching a peak at 6 h and being significantly higher than the control group (*p* < 0.05), the high-concentration group peaked at 12 h, and its MDA content from 6 to 48 h was significantly higher than that of the control group (*p* < 0.05).

### 3.3. Effects of Acute Ammonia Exposure on the Expression of Heat Shock Proteins and Inflammatory Factors in C. alburnus Liver Tissue

**Figure 3:**
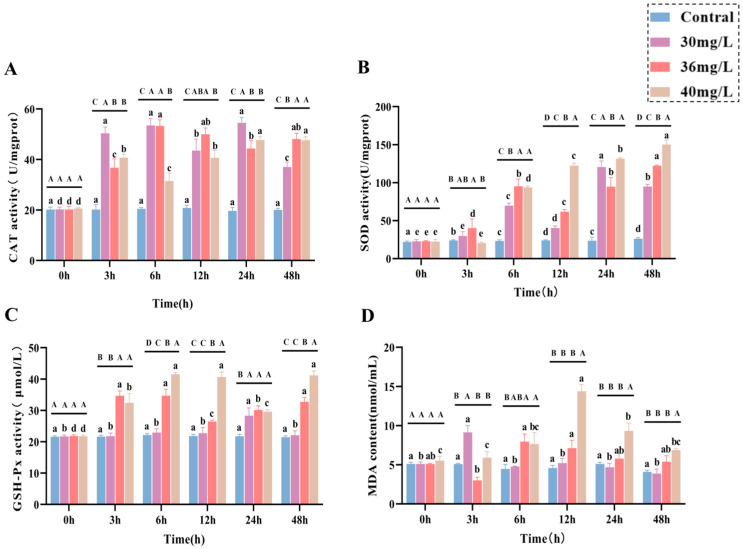
Effect of acute ammonia exposure on the expression of heat shock proteins and inflammatory factors in *C. alburnus* liver tissue. (**A**): *Hsp70*, (**B**): *Hsp90*, (**C**): *IL-10*, (**D**): *TNF-α*. All data are presented as mean ± standard error (*n* = 3 per group). Uppercase letters denote inter-group differences at the same time point (*p* < 0.05), lowercase letters denote intra-group differences at different time points (*p* < 0.05); no same letters indicate significant differences (*p* > 0.05).

The effects of acute ammonia exposure on the expression of heat shock protein genes (*Hsp70* and *Hsp90*) and immunomodulatory genes (*IL-10* and *TNF-α*) in the liver tissues of *C. alburnus* showed distinctive patterns of changes in relation to the concentration and time points. Specifically in the following aspects: for heat shock protein genes, the expression of *Hsp70* (Figure 3A) in the low-concentration group reached the peak at 3 h, while that in the medium-concentration group was significantly higher than that in the control group throughout the exposure period (*p* < 0.05); the expression of *Hsp90* (Figure 3B) in the treated group reached the peak at 3 h, and then showed a decreasing and then increasing trend. For immunomodulatory factors, *IL-10* (Figure 3C) and *TNF-α* (Figure 3D) genes showed a trend of first increasing and then decreasing under low and medium concentration of ammonia exposure; *IL-10* maintained high expression in the low-concentration group, and was significantly higher than the control group in the high-concentration group only at 12 h; *TNF-α* showed a trend of continuous increase in the high-concentration group.

### 3.4. Alterations in Apoptotic Gene Expression and Cellular Apoptosis in the Liver Tissue of C. alburnus After Acute Ammonia Exposure

**Figure 4:**
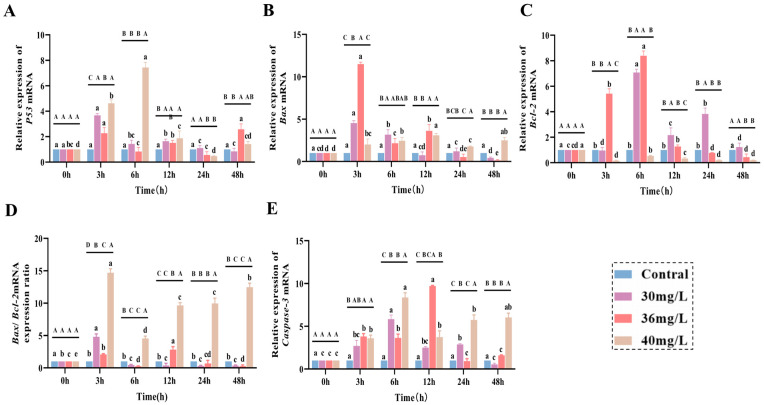
Effect of acute ammonia exposure on the expression of apoptotic genes factors in *C. alburnus* liver tissue. (**A**): *P53*, (**B**): *Bax*, (**C**): *Bcl-2*, (**D**): *Bax/Bcl-2*, (**E**): *Caspase-3*, All data are presented as mean ± standard error (*n* = 3 per group). Uppercase letters denote inter-group differences at the same time point (*p* < 0.05), lowercase letters denote intra-group differences at different time points (*p* < 0.05).

In the liver tissues of *C. alburnus*, the expression levels of apoptotic genes (*P53*, *Bax*, *Bcl-2*, and *Caspase-3*) exhibited distinct variation patterns in response to acute ammonia exposure, which were dependent on both the concentration and the duration of exposure. As for apoptosis genes, the expression of *P53* (Figure 4A) in the treated group showed an overall trend of increasing and then decreasing, and the peak value of the high-concentration group (6 h) was delayed compared with that of the medium and low-concentration groups (3 h); and it was 7.4 times higher than that of the control group. The expression of *Bax* (Figure 4B) in the low and medium-concentration groups showed a tendency of increasing and then fluctuating, and it was significantly higher than that of the control group in the high-concentration group at 3 h (*p* < 0.05). In the high-concentration group, the expression of *Bax* gene fluctuated and was lower than that of the control group, while the expression of *Bcl-2* (Figure 4C) gene increased and then decreased in the low and medium-concentration groups, and reached a peak at 6 h, which was significantly higher than that of the control group (*p* < 0.05). The overall expression of *Bax/Bcl-2* (Figure 4D) was higher in the high-concentration group than in the other treatment groups and was significantly higher than that in the control group throughout the exposure period (*p* < 0.05). *Caspase-3* gene expression was shown in Figure 4E. The expression of *Caspase-3* gene in all treatment groups showed a trend of increasing and then decreasing. There was no significant difference between the low-concentration group and the control group at 3 h, 12 h and 48 h, and there was no significant difference between the medium-concentration group and the control group at 24 h and 48 h. The high-concentration group was significantly higher than the control group at 3–48 h, and the overall expression trend was higher than that of the other treatment groups.

**Figure 5:**
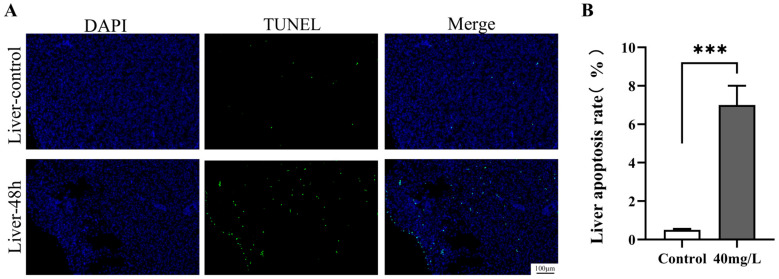
Effect of ammonia stress on the apoptosis of *C. alburnus* liver cells for 48 h. (**A**): Representative images of immunofluorescence, DAPI blue fluorescence for nuclei; TUNEL green fluorescence for apoptotic cells; Merge is a composite graph of DAPI and TUNEL. (**B**): Apoptosis rate of liver tissue cells, *p* < 0.001 is indicated as “***”.

The expression profiles of apoptotic genes indicated that an ammonia concentration of 40 mg/L significantly induced hepatocyte apoptosis. To corroborate the induction of apoptosis, TUNEL assays were conducted on liver tissue samples from the 40 mg/L exposure group at 48 h post-exposure; the results are depicted in Figure 5A. In the control group, the number of TUNEL-positive apoptotic cells in the liver tissue was exceedingly low. Conversely, partial cellular apoptosis was observed in the liver tissue 48 h after exposure to high concentrations of ammonia. As illustrated in Figure 5B, the quantity of TUNEL-positive apoptotic cells in the high-concentration group was significantly elevated compared to the control group (*p* < 0.001).

### 3.5. Histopathological Alterations in the Liver Tissue of C. alburnus Following Acute Ammonia Exposure

**Figure 6:**
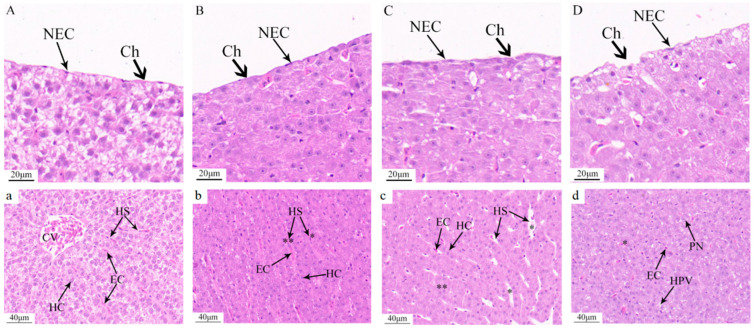
Pathological changes in liver tissue after acute ammonia exposure of *C. alburnus*. Compared to the control group, 30 mg/L. Hyperemia of hepatic sinuses (*p* < 0.01 is indicated as “**”); stenosis of hepatic sinuses (*p* < 0.05 is indicated as “*”); 36 mg/L. Dilated hepatic sinusoids (*p* < 0.05 is indicated as “*”) and blurred hepatocytes (*p* < 0.01 is indicated as “**”); 40 mg/L. Fuzzy structure of hepatic sinusoids (*p* < 0.05 is indicated as “*”). (**A**,**a**): control group; (**B**,**b**): low concentration ammonia stress 48 h (30 mg/L); (**C**,**c**): medium concentration ammonia stress 48 h (36 mg/L); (**D**,**d**): high concentration ammonia stress 48 h (40 mg/L). Ch—chorion, NEC—nucleus of epithelial cell, CV—central vein, HC—hepatocyte, EC—red blood cell, HS—hepatic sinusoid, PN—nuclear migration, HPV—hepatocyte vacuolation.

Following 48 h of acute ammonia exposure, conspicuous histopathological changes were observed in the liver tissue cells of *C. alburnus*. Hepatocytes exhibited morphological alterations and membrane damage that escalated with increasing exposure concentrations. Additionally, hepatocytes became increasingly disorganized, cytoplasmic vacuolization was evident, and the structure of the sinusoids was obliterated. Specifically, in the control group, hepatocyte plasma membranes were intact, epithelial cells were flat, and cell nuclei were clearly discernible (Figure 6A). Hepatocytes displayed a polygonal shape with well-defined contours and were neatly arranged. Cord-like structures were aligned on either side of the hepatic sinusoids, which were of normal size and radially distributed around the central vein (Figure 6a). In the low-concentration group (30 mg/L), both the plasma membrane and epithelial cell nuclei showed no significant alterations compared to the control group (Figure 6B); however, hepatocytes were swollen and enlarged but still maintained a cord-like arrangement. The hepatic sinusoids were noticeably narrowed, and some exhibited congestion (Figure 6b). At the medium concentration (36 mg/L), the plasma membrane thickened, contours became indistinct, and the nuclei of the single-layered flat epithelial cells were unclear (Figure 6C). Hepatocytes were swollen and enlarged, with most maintaining a cord-like arrangement, although a few exhibited ambiguous structures. The hepatic sinusoids were dilated, and red blood cells were reduced (Figure 6c). In the high-concentration group at 48 h (40 mg/L), the plasma membrane exhibited folding and rupture, and significant morphological changes were observed in the epithelial cells (Figure 6D). Hepatocytes were swollen and rounded, displaying hydropic degeneration and pale-staining cytoplasm. Cytoplasmic vacuolization was evident in some hepatocytes, and the cord-like structural arrangement was lost. Most hepatic sinusoids had disappeared. and the remaining few had indistinct structures with reduced red blood cells (Figure 6d).

## 4. Discussion

### 4.1. Ammonia Exposure Alters Hepatic Stress Hormones and Metabolic Indices

In intensive aquaculture systems, a substantial amount of nitrogenous organic matter is readily released into the aquatic environment. If not promptly managed, these substances are rapidly converted into ammonia nitrogen under the action of bacterial decomposition, leading to a significant elevation in waterborne ammonia levels and posing severe detrimental effects on both the aquatic environment and its biota. In environmental biology, aquatic organisms, particularly fish, are often considered vital bioindicators for assessing the health status of aquatic ecosystems [23,24,25]. In the present study, a comparative analysis was conducted between the existing ammonia exposure data for *C. alburnus* and other Cyprinidae fish species [26,27].

Ammonia exposure significantly alters biochemical indices in the blood of aquatic animals, and typical stress-related changes result in significant increases in blood COR, Amm and Glu. Refaey measured blood indices in Amur catfish (*Silurus asotus*) after transport and found that COR levels were significantly increased, reflecting the increased stress levels in the fish during transport, and elevated COR levels acted as a secondary response to transport stress, causing physiological changes in amur catfish, suggesting that the fish develop certain energy demands under transport stress [28]. Plasma COR levels were elevated in rainbow trout (*Oncorhynchus mykiss*), common carp (*Cyprinus carpio*), and goldfish (*Carassius auratus*) under acute ammonia exposure [29]. In the present experiment, COR levels were elevated in all treatment groups at 3 h of ammonia exposure and were significantly higher than those in the control group, suggesting that ammonia exposure at this time caused the activation of the *C. alburnus* HPI axis, which resulted in an initial stress response and elevated COR levels, consistent with findings in other fish species. Plasma ammonia levels were found to be positively correlated with ambient ammonia concentrations in silver sea bream (*Sparus sarba*) and turbot (*Scophthalmus maximus*) exposed to different concentrations of ammonia [30,31]. In multiple experiments with varying exposure concentrations and durations, fish Amm responded to ammonia more rapidly, significantly and consistently than other indicators, thus serving as the most direct and significant physiological indicator of ammonia stress [32]. In this study, all treatment groups showed decreased Amm and increased COR at 3 h, likely due to ammonia-induced initial stress that accelerated ammonia metabolism to mitigate damage and reduce blood ammonia. Serum Amm was significantly higher in all three concentration groups than in the control group from 6 to 24 h. It is likely that the inability to excrete ammonia in a timely manner during the stress adaptation stage resulted in the accumulation of serum Amm.

From 12 to 48 h, Amm decreased in low and medium-concentration groups, indicating *C. alburnus* adaptation to ammonia. However, Amm in the high-concentration group continued to rise and remained significantly higher than the control. This shows *C. alburnus* could adapt to 40 mg/L ammonia, but failed to excrete ammonia efficiently within 48 h at this concentration, causing sustained Amm elevation. High Amm indicated 40 mg/L ammonia exceeded *C. alburnus* stress adaptation capacity and suggested potential organ damage. Endogenous glycogen is the main carbohydrate reserve. Aquatic animals need substantial energy to maintain homeostasis under adverse conditions [33], stimulating inter-renal tissues to produce large amounts of COR for hepatic glycogenolysis [34]. Elevated Glu aids fish in coping with stressors. Ammonia-exposed fish often show enhanced. In the current study, COR was elevated at 3 h in the treatment groups, while Glu began to increase at 6 h. This temporal difference reflects the physiological cascade of the stress response, COR as an upstream stress signal first increases to promote hepatic glycogenolysis and gluconeogenesis, and Glu (as a downstream metabolic product) rises subsequently [35]. Thus, the earlier detection of COR is a result of the sequential order of the stress response, rather than higher sensitivity compared to Glu.

### 4.2. Ammonia Exposure Induces Hepatic Tissue Damage in C. alburnus

ALT and AST are aminotransferases in the liver, which are often used as indicators of acute liver injury, as the concentration of these enzymes is elevated in the blood when hepatocytes are damaged. Exposure of zebrafish (*Danio rerio*) to the test compounds and the subsequent increase in serum AST and ALT activities indicated that zebrafish hepatocytes were damaged by such exposure [36]. Elevated blood ALT and AST activities were observed in carp and Nile tilapia (*Oreochromis niloticus*) following ammonia exposure [37,38]. suggested that ammonia toxicity is related to oxidative stress and that oxidative damage may be one of the important pathways by which ammonia induces organ damage. In this study, both ALT and AST were significantly higher in the treated group than in the control group at 6–48 h of ammonia exposure, suggesting that all ammonia exposure treatment concentrations caused acute liver injury.

Since the liver is an important detoxification organ in fish, the toxic effects of chemical substances usually initially manifest in the liver. In this experiment, the increased activities of serum ALT and AST indicated acute injury to liver tissue. Pathological sections showed that as the ammonia concentration increased, the serous membrane covering the liver tissue was damaged; acute exposure led to changes in hepatic parenchymal cells. Specifically, the morphology of hepatocytes changed from polyhedral to round, their outlines shifted from clear to blurred, and their cord-like arrangement became disordered. Observation of hepatocytes of *C. alburnus* via transmission electron microscopy revealed that their cytoplasm contained abundant mitochondria, ribosomes, well-developed endoplasmic reticulum, numerous lysosomes, and glycogen granules, with large and distinct nucleoli [39]. These characteristics indicate that the material and energy metabolism in the hepatocytes of *C. alburnus* is active. In this study, the increased volume of hepatocytes under low-concentration ammonia stress likely reflects an increase in mitochondria, endoplasmic reticulum, ribosomes, and lysosomes in the cytoplasm. This is presumably caused by increased protein synthesis induced by environmental changes, which in turn leads to cytoplasmic swelling. Some studies suggest that the presence of round vacuoles of varying sizes in the hepatocyte cytoplasm may be associated with the loss of glycogen substances [40]. Given that the hepatocytes of *C. alburnus* themselves contain abundant glycogen, it is inferred that the vacuolation of hepatocytes observed in this study may result from the release of hepatic glycogen into the bloodstream under ammonia stress. This release elevates blood glucose to meet the energy requirements of the stress response, ultimately leading to hepatic glycogen vacuolation. Other studies indicate that changes in cell morphology may reflect the disintegration of polymeric proteins, which are components of the cytoskeleton [41]. Under high-concentration ammonia stress, hepatocytes undergo hydropic degeneration, which reflects that ammonia stress causes certain damage to liver tissue. Observation of the liver of *O. niloticus* exposed to low-concentration ammonia stress for 42 days showed cloudy swelling and hydropic degeneration of hepatocytes; such damage is considered to indicate a disorder of energy metabolism and an alteration of cell function [42]. Additionally, after 96 h of acute ammonia stress, star sturgeon (*Acipenser stellatus*) exhibited hepatocyte steatosis and cloudy swelling [43], and the pathological sections of this experiment also showed similar results.

### 4.3. Ammonia Exposure Triggers Hepatic Oxidative Stress

Ammonia exposure induces oxidative stress by disrupting oxidative function, leading to excessive ROS production in fish [44]. Studies on bighead carp (*Aristichthys nobilis*) demonstrated that ammonia exposure increases ROS production and antioxidant enzyme gene expression, triggering oxidative stress, immunosuppression, inflammation, and stress responses. Following 96 h of ammonia exposure, liver CAT levels in *C. carpio* increased significantly, indicating that CAT modulates oxidative homeostasis by adjusting activity to counter oxidative stress [45]. Ammonia intoxication upregulated SOD and GSH-Px levels in liver tissue of banded catfish (*Tachysurus fulvidraco*) [46]. Acute 3 h ammonia exposure in carp significantly elevated liver SOD, CAT, and GSH-Px [47]. Consistent with these findings, in the present study, SOD and CAT were significantly higher in all treatment groups than the control during 3–48 h of ammonia exposure, with fish producing substantial antioxidant enzymes to scavenge excess ROS and mitigate oxidative damage. Increased antioxidant enzyme activity is linked to heightened oxidative stress and free radical activity, with more intense stress driving greater enzyme production [48]. For GSH-Px, the low-concentration group showed minor changes during exposure, while medium and high-concentration groups exhibited fluctuations at elevated levels, with all groups significantly higher than the control at 48 h. By the 48 h mark, a clear positive correlation emerged between ammonia concentration and SOD activity, indicating a time- and dose-dependent escalation of oxidative stress. This progressive intensification of the antioxidant response mirrors the temporal pattern previously documented in *C. carpio* under ammonia duress [37]. The dynamics of MDA content revealed a concentration-specific response. While an initial rise in MDA signaled early oxidative damage across all treatment groups, the timing of the peak and subsequent recovery trajectory diverged markedly [49]. At lower concentrations (≤36 mg/L), MDA levels crested earlier (3–6 h) before declining, pointing to a successful mobilization of the hepatic antioxidant system that mitigated damage and facilitated recovery. A parallel adaptive response has been reported in yellow catfish (*Pelteobagrus fulvidraco*) under sublethal toxicant stress [46]. In stark contrast, exposure to 40 mg/L ammonia provoked a different scenario: the MDA peak was not only delayed until 12 h but also reached a magnitude 3.14 times that of the control group. Furthermore, these elevated levels persisted throughout the 48 h exposure. This pattern of delayed yet sustained lipid peroxidation finds a counterpart in the intestines of Asian clams (*Corbicula fluminea*) under severe ammonia stress [50]. Such persistence indicates that the 40 mg/L concentration overwhelmed the defensive capacity of *C. alburnus*, inflicting severe and irreversible damage on hepatocytes. This observation underscores a critical finding: a transient boost in antioxidant enzyme activity is often inadequate to fully avert cellular damage under intense oxidative stress [50], a conclusion corroborated by studies on *P. fulvidraco* [51].

### 4.4. Ammonia Exposure Modulates Hepatic Stress and Inflammatory Responses

#### 4.4.1. Heat Shock Protein (*Hsp*) Regulation

Heat shock proteins (*Hsp*) are highly sensitive to the intensity of the stressor and the duration of exposure and are major biomarkers for assessing environmental stress. Ammonia exposure induces elevated *Hsp70* and *Hsp90* expression in Scleractinian fish [52,53]. However, differential expression of *Hsp70* and *Hsp90* was also observed in ammonia exposure, and both hepatopancreatic *Hsp70* and *Hsp90* expression were significantly increased and differentially expressed under acute ammonia exposure to shrimp for 72 h [54]. Ammonia exposure induced elevated *Hsp70* and *Hsp90* expression in pufferfish (*Tetraodontidae*), with similar levels of response in the three concentration groups in *Hsp70*, but a strong response in the low-concentration group in *Hsp90* [55]. Elevated COR inhibits tilapia (*Oreochromis mossambicus*) hepatic *Hsp70* expression [56]. Studies on COR and heat shock proteins have revealed distinct patterns. For *Hsp70*, elevated COR levels were associated with opposing trends between *Hsp70* mRNA expression and its protein product. This phenomenon suggests that translation of pre-existing or newly synthesized mRNAs may block gene activation [57]. *Hsp90* exhibits a different regulatory pattern: COR first up-regulates the *Hsp90* gene, and the synthesized *Hsp90* protein then down-regulates the gene [58,59]. These findings align with the results of the present study. Tissue damage is frequently accompanied by inflammation. Inflammation serves as an organismal defense response to external stimuli. It facilitates the repair of damaged tissues by regulating a complex network of cytokines within the organism [60]. Tumor necrosis *TNF-α* is a pro-inflammatory factor secreted by macrophages. It plays a central role in mediating the inflammatory response [61].

#### 4.4.2. Inflammatory Cytokine Responses

It has been shown that ammonia exposure produces inflammation, and rainbow trout induces *TNF-α* expression early in ammonia exposure to produce an inflammatory response [62], which down-regulates a variety of cytokines for organismal defense. Similar results were obtained in this experiment. Elevated *TNF-α* expression at 3–6 h of exposure induced an inflammatory response in the treatment groups, at which time the expression of the anti-inflammatory factor *IL-10* was suppressed at the high concentration, while the opposite was observed in the other treatment groups, indicating that 40 mg/L ammonia inhibited the anti-inflammatory response of *C. alburnu* and induced inflammation during the stress period of ammonia exposure; with increasing exposure time, the medium and low-concentration groups reduced the expression of inflammatory factors by increasing anti-inflammatory factors, adapted to the stress and restored homeostasis, in contrast to the high-concentration group, where the 40 mg/L ammonia concentration induced a sustained increase in the inflammatory response in *C. alburnus*.

### 4.5. Ammonia Exposure Activates Hepatic Apoptosis via the P53-Bax/Bcl-Caspase Pathway

It has been shown that excess ROS produced in fish impairs mitochondrial membrane permeability and leads to apoptosis [63]. Apoptosis is a natural programmed mode of cell death regulated by genes [64] and is a defense mechanism of fish against external aggression, as well as an important pathway for the removal of damaged cells and the maintenance of homeostasis [65]. The *P53* gene can induce apoptosis by disrupting the fidelity of DNA replication and cell division in response to cellular stress. In the present study, *P53* expression was activated in all treatment groups at 3 h and then decreased in the low and medium-concentration groups but continued to decrease in the high-concentration group at 12 h, which lasted for a longer period of time than in the other two groups. Up-regulation of *P53* triggered the up-regulation of the apoptotic gene *Bax* and the inhibition of the expression of the anti-apoptotic gene *Bcl-2*. In the present study, the *Bax*/*Bcl-2* expression ratio of all treatment groups increased at 3 h of ammonia exposure, which induced hepatocyte apoptosis; the expression of apoptotic genes was restored after 12 h of ammonia exposure up to 36 mg/L, which indicated that the low and medium-concentration groups produced stress adaptation and down-regulated apoptotic genes to restore the homeostasis of the fish body after 12 h of exposure; whereas, the 40 mg/L ammonia exposure continued to induce the up-regulation of *Bax*/*Bcl-2* expression after the elevation of *P53*, which lasted for a longer period than the other two groups. After 40 mg/L ammonia exposure triggered the elevation of *P53*, it continued to cause the elevation of *Bax*/*Bcl-2* expression level and the activation of mitochondria-dominated *P53-Bax*/*Bcl-2* endogenous apoptosis in the fish, while the caspase-dependent apoptotic pathway was activated in the fish during the *Caspase-3* exposure, which was significantly higher than that in the control group. On the basis of the gene expression assay, in order to verify the production of apoptosis in the 40 mg/L group, a further TUNEL fluorescence assay was performed, and the results confirmed the production of hepatocyte apoptosis in the 40 mg/L ammonia exposure for 48 h, and the positive apoptotic cells were extremely significantly higher than those in the control group. These suggest that *C. alburnus* may induce hepatocyte apoptosis through the *P53-Bax*/*Bcl-Caspase* complex apoptotic signaling pathway under 40 mg/L ammonia exposure.

## 5. Conclusions

This study systematically investigated the impact of different exposure concentrations on the stress responses and physiological regulation in *C. alburnus*, offering precise biomarkers for environmental monitoring and contributing to the establishment of more scientifically rigorous water quality standards. The liver is identified as a key target organ affected by acute ammonia exposure in this study and serves as a practical indicator for tracking ammonia-induced toxic responses in aquatic environments. Within the range of 30 mg/L to 40 mg/L ammonia concentrations, *C. alburnus* exhibited varying degrees of hepatic injury and stress responses. Similarly, the study revealed that the fish employed multiple physiological pathways for adaptation to different levels of ammonia exposure, including antioxidant enzyme activity, ammonia metabolism, and inflammatory responses. At higher concentrations, activation of specific signaling pathways was observed, notably the *P53-Bax*/*Bcl-Caspase3* pathway leading to hepatocyte apoptosis. These findings not only offer new insights into the ecotoxicology of ammonia pollution in aquatic environments but also underscore the liver’s significance as a sensitive indicator organ for ammonia-induced stress and toxic responses in this study. Future research should further explore the universality and specific impacts of these physiological regulatory mechanisms across different aquatic environments and fish species.

## Figures and Tables

**Table 1:** Target gene RT-PCR primers.

Accession No.	Amplicon Size (bp)	Gene	Primer	Primer Efficiency (%)	R^2^
XM_042754349.1	134	*P53* F	GCCCATCCTCACAATCATCACTCTG	96.7	0.996
		*P53* R	CCTGGTCTTTCCTGAAGTTGCTCTC	96.7	0.996
KJ174685.1	135	*Bax* F	TCTACTTTGCGTGTCGGCTTGTC	102.2	0.998
		*Bax* R	TCCATCCACCCTGTTCCCTGATC	102.2	0.998
XM_019067767.2	131	*Bcl-2* F	CGAGTTTGGAGGGACCGTTTGTG	94.5	0.995
		*Bcl-2* R	AGCCGCCGTTCTCCTGGATC	94.5	0.995
XM_042755485.1	128	*TNF-α* F	GACCAGAACAACAGGGAGCACATC	98.2	0.999
		*TNF-α* R	AGTTTGGAGAGAGGGCAGAAGAGAG	98.2	0.999
XM_042766262.1	80	*IL-10* F	TCTCAAGCGGGATATGCGGAAATG	101.6	0.999
		*IL-10* R	ACGAGTTCTTTATGCTGGCGATCTC	101.6	0.999
JN544930.1	137	*Hsp70* F	CACCAATGACAAGGGCAGACTGAG	95.8	0.995
		*Hsp70* R	TGTTGAAGGCGTAGGACTCCAGAG	95.8	0.995
XM_042721808.1	104	*Hsp90* F	AACTCACGGACACCAAAGCATACG	101.9	0.998
		*Hsp90* R	CTCCTCTTCTGGCTCTTCCTCTACC	101.9	0.998
KF055462.1	81	*Caspase-3* F	ACACAACAGATGCTGGTAAGGATGG	99.3	0.996
		*Caspase-3* R	GACTGTACCTGTAGGCATGTGACTG	99.3	0.996

**Table 2:** Total ammonia concentration and number of dead individuals in the experiment.

TAN (mg/L) *	CNH_3_ (mg/L) *	Total Amount	Total Deaths	CMR (%) *
26.68	1.89	30	3	10
30.94	2.19	30	8	26.67
35.89	2.54	30	15	50
41.63	2.95	30	21	70
48.28	3.42	30	28	93.33

* TAN: total ammonia nitrogen; CNH_3_: non-ionic ammonia; CMR: Cumulative Mortality Rate.

## Data Availability

The original contributions presented in this study are included in the article. Further inquiries can be directed to the corresponding authors.
